# Association of Metformin Treatment with Risk for Death in Diabetic Patients with Concomitant Gastric Cancer

**DOI:** 10.3390/cancers15164134

**Published:** 2023-08-16

**Authors:** Jae-Hong Joo, Hyun-Soo Zhang, Jiyeon Chun, Eun-Cheol Park, Sohee Park

**Affiliations:** 1 Department of Public Health, Graduate School, Yonsei University, Seoul 03722, Republic of Korea; jhj3040@yuhs.ac (J.-H.J.); ecpark@yuhs.ac (E.-C.P.); 2 Institute of Health Services Research, Yonsei University, Seoul 03722, Republic of Korea; 3 Department of Biostatistics, Graduate School of Public Health, Yonsei University, Seoul 03722, Republic of Korea

**Keywords:** metformin, gastric cancer, diabetes mellitus, all-cause death

## Abstract

**Simple Summary:**

During the 5-year follow-up within the study period, the cumulative incidence of all-cause death was notably lower in the metformin treatment group compared to the non-treatment group (27.5% vs. 32.8%). The analysis further revealed a significantly reduced hazard ratio (HR) for all-cause death in the metformin treatment group (HR: 0.80, 95% CI 0.78–0.82). This population-based cohort study provides evidence that long-term metformin treatment is associated with a decreased risk of mortality among individuals who have both diabetes and gastric cancer.

**Abstract:**

Importance: Despite the existing guideline’s recommendation of metformin therapy as the initial approach for managing diabetes mellitus (DM), there remains a scarcity of comprehensive documentation regarding metformin’s impact on outcomes that are important for patients. Objectives: The objective of this study was to assess the potential impact of metformin treatment on the risk of death in individuals diagnosed with both gastric cancer and pre-existing diabetes mellitus (DM); Design, Setting, and Participants: The study made use of a dataset encompassing nationwide health insurance claims, allowing for a retrospective analysis of all patients with a history of gastric cancer diagnosis (classified under International Classification of Diseases 10th Revision code: C16.X) spanning from 1 January 2002 to 31 December 2012. The primary objective was to observe death within a 5-year follow-up period. The study population comprised 63,664 individuals who fell into two categories: those treated with metformin (n = 29,548) and those who did not receive metformin treatment (n = 34,116). This classification was based on the initial treatment allocation following the diagnosis of gastric cancer. Exposures: Metformin treatment, comorbidities, concurrent medication, and procedural information. Outcomes: All-cause death, disease-specific death, cardiovascular death. Results: During the 5-year follow-up period, the metformin treatment group exhibited a lower cumulative incidence of all-cause death (27.5%) in comparison to the group not receiving metformin treatment (32.8%). Furthermore, the relative hazards for all-cause death were significantly reduced in the metformin treatment group (HR: 0.80, 95% CI 0.78–0.82), indicating a lower risk of death when compared to the non-metformin group. In addition, metformin treatment was associated with lower occurrences of disease-specific death (related to gastric cancer) and cardiovascular death when compared to the group not undergoing metformin treatment. Conclusions: The findings demonstrated that the use of metformin was effective at improving prognosis among gastric cancer patients documented with prior DM. In this population-based cohort study, metformin treatment was associated with reduced risk of mortality.

## 1. Introduction

Malignant neoplasm is the leading cause of death worldwide, contributing to approximately 19.3 million new cancer cases and 10 million deaths in 2020 [1]. Particularly, gastric cancer ranked as the fifth most common cancer, with 1,089,103 newly diagnosed cases globally, accounting for 768,793 deaths. The highest incidence rate was in Eastern Asia, with 32.5 cases per 100,000 persons, followed by Eastern Europe (17.4 per 100,000) [2]. Gastric cancer has been one of the leading diagnosed cancers and causes of cancer-related mortality in South Korea, but it has shown a continuous declining trend over the past two decades [3,4]. Consistent gains in survival are expected in the future due to recent advances in early detection and cancer treatment [5,6].

Diabetic patients with concurrent gastric issues are considered to be at an elevated risk of future mortality and morbidity [7]. Hyperinsulinemia and chronic inflammation, both associated with diabetes, play significant roles in the neoplastic process. As a result, cancer patients with diabetes require interventions to reduce blood glucose levels and enhance insulin sensitivity [8].

**Figure 1:**
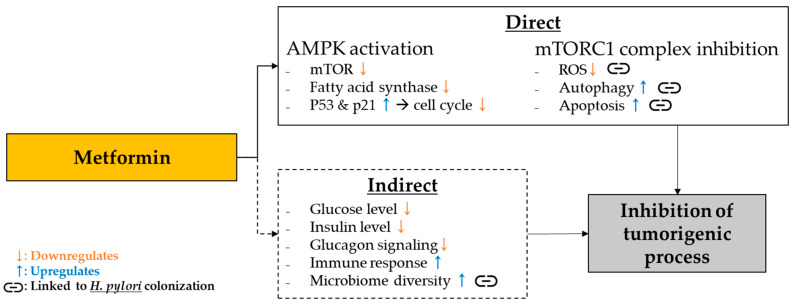
Summary of metformin mechanisms of action and gastric cancer.

According to current guidelines provided by the American Diabetes Association (ADA) and the European Association for the Study of Diabetes (EASD), metformin is recommended as the primary oral therapeutic approach for managing diabetes. Metformin is a widely utilized medication that offers evident advantages in terms of glucose metabolism [9]. The prognostic significance of survival outcomes for diabetic patients has been evaluated in preclinical studies [10,11,12]. Furthermore, metformin operates through the 5′-adenosine monophosphate-activated protein kinase (AMPK) mechanism, which modulates carcinogenesis [13]. Metformin’s anti-tumor effects also involve inhibiting mitochondrial complex 1 (mTORC1), allowing it to decrease the generation of reactive oxygen species (ROS) and trigger autophagy while fostering diversity in the gastric microbiome (refer to Figure 1) [13,14]. Decreased microbiome diversity is closely associated with H. pylori colonization, consistently implicating metformin’s underlying mechanism in managing gastric cancer [14].

However, direct evidence that comprehensively supports the efficacy of metformin in patients with both concurrent diabetes mellitus (DM) and gastric cancer is still lacking. Physiologically, metformin has been shown to reduce glucose production and potentially have an anticancer effect [15]. Nevertheless, not all of its effects can be adequately elucidated due to the complexity and incomplete understanding of the underlying mechanism of action.

In the context of gastric cancer, metformin has recently garnered attention as a potential treatment with anticancer properties [16]. Moreover, there has been a renewed interest in the drug’s impact on diabetic patients with gastric cancer [12]. Therefore, the aim of this study was to assess the potential effect of metformin treatment on the risk of death among patients with gastric cancer who have previously been diagnosed with diabetes mellitus.

## 2. Method

### 2.1. Study Design, Data Source and Study Population

The study was based on the National Health Insurance Service (NHIS) database. The NHIS is a nonprofit and single-payer system generally administered by the Ministry of Health and Welfare. This study was conducted according to the Declaration of Helsinki and approved by the independent Institutional Review Board of Yonsei University Health System (IRB 4-2021-0374) with no written informed consent because patients’ records/information was anonymized prior to analysis. The data, comprising health insurance claims for the nationwide population, were utilized to retrospectively examine all patients with a history of gastric cancer diagnosis using the International Classification of Diseases 10th (ICD-10) code corresponding to ‘C16.X’ from 1 January 2002 to 31 December 2012. Then, the study was designed to observe the occurrence of death at a 5 year follow-up period.

The total of 281,906 newly diagnosed gastric cancer patients was derived from the NHIS claims data after a 1-year washout period, fitting well with the number reported by Korean National Cancer Registration (KNCR) Statistics. From the selected study population, patients with missing demographic information (n = 1119) and patients with multiple cancers (n = 40,683) were excluded. Accordingly, 240,104 gastric cancer patients were eligible for the analysis between 2003 and 2012.

Of the eligible gastric cancer patients, 154,640 patients with no prior history of DM were excluded. Additionally, those with less than 30 days of follow-up duration (n = 19,495) were excluded.

**Figure 2:**
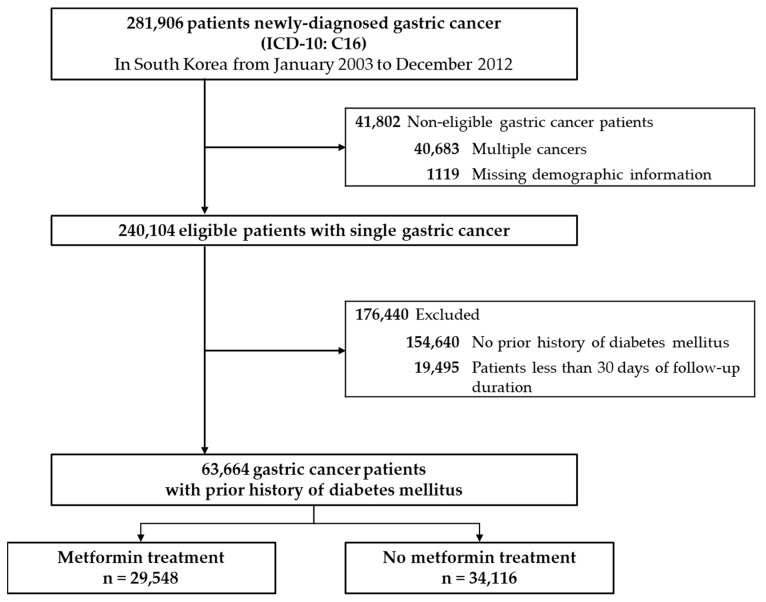
Exclusion criteria of study subjects.

Consequently, this study comprised the remaining 63,664 patients who were treated with metformin (n = 29,548) and who were never treated with metformin (n = 34,116) during the initial treatment allocation after a gastric cancer diagnosis (Figure 2).

### 2.2. Study Outcomes

All-cause death is determined based on death certificates obtained from the NHIS (National Health Insurance Service) database. Disease-specific death was defined using the Tenth Revision of the International Classification of Diseases (ICD-10) codes corresponding to ‘C16.X’, which were recorded on the death certificates.

In light of metformin’s impact on low-density lipoprotein (LDL) cholesterol and its potential to stimulate cholesterol transport, this study also incorporated cardiovascular death as one of the outcome measures [17]. Cardiovascular death cases were identified via the National Statistical Office of Korea, which provided death certificates with a precision rate of 92% for specific causes of death [18,19,20]. Cardiovascular death was identified by a death certificate containing at least one diagnosis related to cardiovascular conditions (such as acute myocardial infarction, stroke, heart failure, or sudden cardiac death). A detailed explanation of each clinical outcome is presented in Table S1.

### 2.3. Prior History of DM

DM was identified through the ICD-10 code recorded in the claims data. Patients who received a diagnosis with the ‘E10.X-E14.X’ code in their claims records prior to the diagnosis of gastric cancer were categorized as having prior DM. The duration of DM prior to the gastric cancer diagnosis was 4.48 years (SD: 3.09) in the metformin treatment group and 4.53 years (SD: 3.11) in the no-treatment group. An analysis of variance (ANOVA) was conducted to test for statistically significant differences in the duration of diabetes between these two groups, and no significant difference was found (*p*-value = 0.721).

The severity of DM was assessed based on the patients’ baseline comorbidities and concurrent medication usage. Comorbidities taken into account included hypertension, chronic kidney disease, and the Charlson comorbidity index. A comprehensive list of comorbidities considered in this study is presented in Table S2. Concurrent medications encompassed the utilization of insulin, as well as oral hypoglycemic agents (OHAs) such as Sulfonylurea, Biguanide, Thiazolidinedione, and SGLT-2 inhibitors.

### 2.4. Treatment Allocation

The allocation of treatment was determined based on the number of days metformin was prescribed during the first year following the diagnosis of gastric cancer. Among the final sample of 63,664 patients, 29,548 (46.4%) were prescribed metformin for 365 days, while 29,852 (46.9%) had no metformin prescription. The remaining 4264 patients (6.7%) in the final sample received less than 90 days of prescription during the first year. These patients with limited prescription days were included in the no-treatment group to prevent selection bias [21], as removing this group could lead to a non-representative study sample.

**Figure 3:**
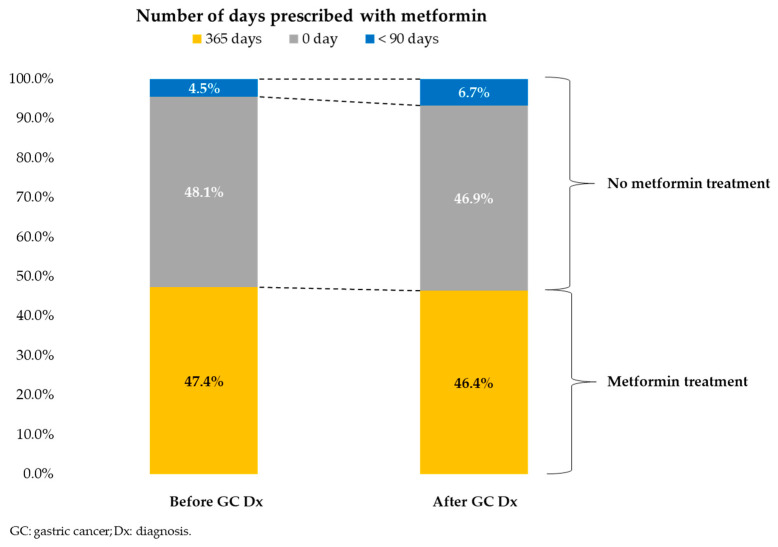
Summary of treatment allocation.

Furthermore, the validity of the treatment allocation was assessed by comparing the number of days metformin was prescribed during the first year with the number of days it was prescribed before the cancer diagnosis. Among patients who had received metformin treatment prior to their gastric cancer diagnosis (47.4%), 46.4% continued to receive metformin during the first year (*p* < 0.001) (refer to Figure 3).

This study followed an intention-to-treat (ITT) approach, commonly employed in randomized clinical trials (RCTs), which involves the inclusion of all patients in the final analysis. Consequently, the treatment allocation established at baseline remains a fixed exposure (treated vs. not treated) for the patients, and subsequent changes in metformin usage are not taken into account. Excluding noncompliant patients could introduce prognostic imbalance among the treatment groups [21].

### 2.5. Statistical Analysis

Continuous variables were expressed as mean ± standard deviation, while dichotomous variables were presented as frequencies and percentages. The estimation of inverse probability of treatment weights (IPTWs) was accomplished using the propensity score, which was determined through logistic regression involving baseline factors such as age, sex, comorbidities, concurrent medication, procedural information, and the year of gastric cancer diagnosis. This approach aimed to minimize confounding bias. In retrospective observational studies based on historical data, many baseline covariates at the subject level are likely to impact both the exposure and the outcome [22]. Thus, including all measured baseline characteristics in the propensity model is safe. Our study selected relevant covariates from the data to reduce confounding bias and constructed an elaborate model to satisfy the strong ignorable condition [23,24,25].

**Figure 4:**
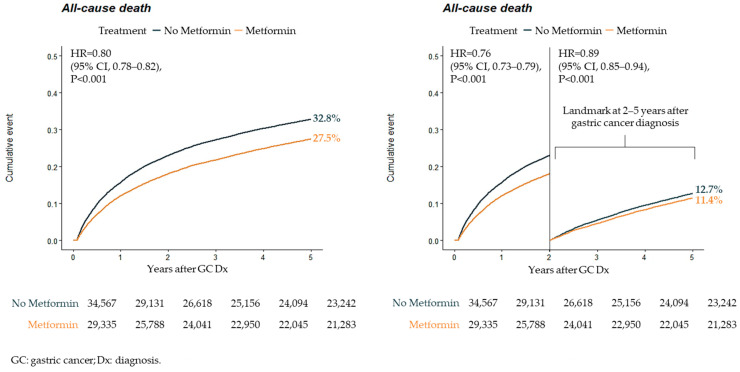
Time-to-event curve for all-cause death.

To stabilize the IPTW, it was multiplied by the marginal probability of receiving each treatment. The imbalance between the two groups for baseline comorbidities and medications was assessed by calculating the difference in effect size using standardized mean difference and visualized using Kernel density plots (refer to Table 1 and Figure S1). Values of standardized mean difference exceeding 0.1 were considered indicative of the potential imbalance between the two groups. Survival rates over a 5-year follow-up period were graphically depicted using the Kaplan–Meier method (refer to Figure 4). The adjusted hazard ratio (HR) for each study outcome was calculated using the Cox proportional hazard regression model with a sandwich variance estimate. Specifically, a cause-specific hazard model was applied to account for death as a competing risk when comparing the incidences of all-cause, disease-specific, and cardiovascular death.

Landmark analysis was employed to address immortal bias. Participants who were right-censored due to early death before reaching the landmark time were not included in the calculation of risk for death [26]. Furthermore, the treatment assignment for the remaining participants was determined based only on data available up to the specific landmark time. Consequently, the dynamic nature of treatment allocation over time was effectively controlled [26,27]. In this study, the landmark time was precisely defined as the period spanning 2 to 5 years following the diagnosis of gastric cancer.

The average survival duration of untreated gastric cancer patients across TNM stages has been reported as 63 months for T1, 25 months for T2, 13 months for T3, and 10 months for T4 [28]. Additionally, T1 gastric cancer has been noted to represent the highest proportion among all gastric TNM stages in South Korea (accounting for 63.9% in 2019) [29]. Selecting a landmark time within the range of 2 to 5 years allows for the exclusion of cases of early death, thereby enabling the estimation of the long-term impact of metformin treatment.

To ensure the robustness of the primary findings, several sensitivity analyses were performed, including the utilization of a time-dependent Cox proportional hazard model and conducting a per-protocol analysis. The time-dependent Cox proportional hazard model takes into account fluctuations in treatment status. In this context, if a patient undergoing treatment ceases to receive prescriptions for a period of 60 days during the study duration, this interval is treated as a period of being unexposed [30]. On the other hand, the per-protocol analysis involves the exclusion of non-compliant patients, which stands in contrast to the intention-to-treat (ITT) approach [27].

To determine statistical significance, a two-sided *p*-value of less than 0.05 was considered as the threshold. All statistical analyses were carried out using SAS version 9.4 (SAS Institute, Cary, NC, USA) and R version 4.0 (The R Foundation, www.R-project.org, accessed on 10 June 2023).

## 3. Results

The patients were followed for an average of 4.1 ± 1.7 years in the metformin treatment group and 4.0 ± 1.7 years in the no-metformin group. The baseline characteristics of the study population are presented in Table 1 both before and after applying stabilized IPTW. These characteristics encompass health-related behaviors (smoking, drinking), comorbidities, concurrent medication, procedural information, and the year of diagnosis. Following the application of stabilized IPTW, the study included 63,902 patients who had been diagnosed with gastric cancer and documented prior DM: 29,335 patients receiving metformin treatment and 34,567 patients not receiving metformin treatment. Post-application of stabilized IPTW, no disparities were observed in baseline comorbidities, concurrent medication, or procedural information (with standardized mean differences < 0.1, as indicated in Table 1 and Figure S1).

Table 2 presents the relative hazards for (1) all-cause death, (2) disease-specific death (as documented by ICD-10: C16), and (3) cardiovascular death. Over the 5-year follow-up period, the incidence of all-cause death was significantly lower in the metformin treatment group (HR: 0.80, 95% CI 0.78–0.82). In comparison to the no-metformin group, metformin treatment was associated with reduced rates of disease-specific death and cardiovascular death.

Figure 4 illustrates the cumulative incidence of all-cause death. The occurrence of all-cause death was notably lower in the metformin treatment group (27.5% vs. 32.8%). During the 2–5 year landmark period after gastric cancer diagnosis, the cumulative incidence and relative hazards of all-cause death were markedly reduced in the metformin treatment group (11.4% vs. 12.7%, HR: 0.89, 95% CI 0.85–0.94).

The results of sensitivity analyses are provided in Table S3, including outcomes from both the time-dependent Cox proportional hazard model and the per-protocol population model. These models consistently demonstrated a significant association between metformin treatment and a reduced risk of death. Specifically, the time-dependent Cox model indicated an HR of 0.78 (95% CI 0.77–0.79), while the per-protocol model showed an HR of 0.63 (95% CI 0.60–0.64).

**Figure 5:**
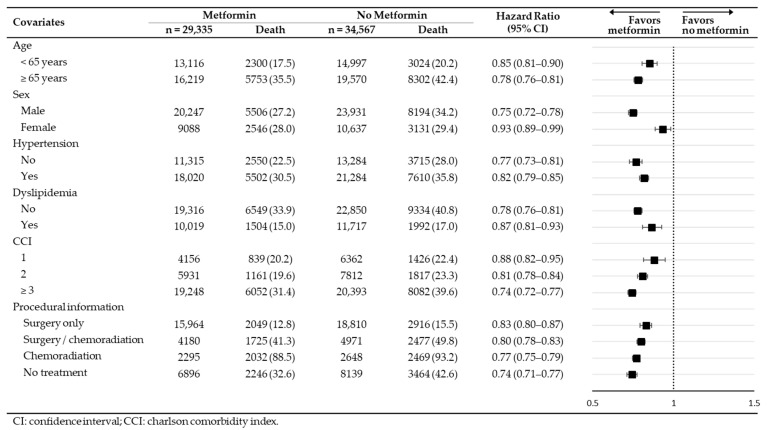
Result of subgroup analysis for all-cause death.

Figure 5 displays the outcomes of subgroup analysis, stratified based on baseline characteristics. Interestingly, there was no significant difference in the treatment effect when the study population was categorized by age, sex, comorbidities, and procedural information. The link between metformin treatment and a reduced risk of all-cause death remained consistent across various baseline characteristics, demonstrating its independence after the application of stabilized IPTW.

Figures S2 and S3 depict the cumulative incidence of disease-specific death and cardiovascular death, respectively. Notably, the incidence of these events was notably lower in the metformin treatment group compared to the non-treatment group (disease-specific death: 19.7% vs. 24.0%, cardiovascular death: 4.5% vs. 5.0%).

## 4. Discussion

The results of the study revealed that the utilization of metformin had a positive impact on the prognosis of gastric cancer patients with a prior diagnosis of diabetes mellitus (DM). Within this population-based cohort study, extended metformin treatment was linked to a decreased risk of multiple outcomes, including (1) all-cause death, (2) disease-specific death related to gastric cancer, and (3) cardiovascular death. These findings collectively suggest that metformin treatment holds the potential to lower the incidence of different types of mortality.

Despite the existing guideline’s recommendation of metformin therapy as the initial approach for managing diabetes mellitus (DM), there remains a scarcity of comprehensive documentation regarding metformin’s impact on patient-relevant outcomes. Despite its availability for over 60 years [31], only a limited number of randomized clinical trials (RCTs) have delved into the efficacy of metformin concerning a wide range of prognostic factors. A recent meta-analysis involving 13 RCTs indicated a potential reduction in all-cause mortality [OR: 0.80; CI 0.60–1.07] when comparing metformin to no therapy [32]. However, it is important to acknowledge that the strength of this evidence is relatively modest due to the restricted sample size of the eligible RCTs.

In contrast, the present nationwide cohort study included 29,548 gastric cancer patients with DM who received metformin treatment—approximately ten times the enrolment of previous RCTs (with enrollments ranging from 174 to 2895). Consequently, this study showcased a markedly improved statistical power, enabling a more robust demonstration of the favorable effect of metformin treatment in comparison to no treatment, particularly concerning all-cause death.

Patients diagnosed with diabetes mellitus (DM) are at a heightened risk of experiencing cardiovascular events, and those with concurrent DM and gastric cancer face an even greater risk of mortality. Consequently, this specific population necessitates a well-structured treatment regimen aimed at reducing glucose levels and preventing adverse cardiovascular outcomes. The cardiovascular safety of metformin has been a topic of discussion since the publication of the groundbreaking UK Prospective Diabetes Study-34 (UKPDS-34) trial in 1998. The UKPDS study demonstrated that early intensive glycemic control using metformin is associated with a lowered risk of diabetes-related complications and mortality [33]. Building upon this significant finding, several clinical practice guidelines, such as those from the American Diabetes Association (ADA) and the European Association for the Study of Diabetes (EASD), recommend metformin as the initial therapeutic choice [9].

The glucose-lowering effects of metformin primarily involve inhibiting hepatic gluconeogenesis and enhancing insulin sensitivity in musculoskeletal tissues. The mechanisms of metformin action are multifaceted, encompassing both AMPK-dependent and AMPK-independent pathways. These include inhibiting mitochondrial respiration, restraining mitochondrial glycerophosphate dehydrogenase activity, and involving lysosomal mechanisms [34,35,36,37]. Metformin’s influence on improving glycemia is primarily attributed to AMPK activation in the liver, although its effects extend to a more intricate network of actions [37]. Despite this progress in understanding, further research is essential to fully grasp how the drug operates within its target population.

Regarding gastric cancer, metformin has recently gained attention as a potential treatment with anticancer properties [14,16]. The actions of metformin and its potential to mitigate the risk of gastric cancer have been highlighted in a recent study by Lan et al. Notably, metformin, which is commonly used to manage diabetes, is associated with favorable effects in cancer prevention due to its therapeutic anti-tumor mechanisms [14]. This has sparked renewed interest in investigating the impact of metformin on diabetic patients diagnosed with gastric cancer [12].

A randomized clinical trial (RCT) conducted by Lee et al. demonstrated that in gastric cancer patients with diabetes, an extended cumulative duration of metformin usage was linked to decreased rates of all-cause mortality and cancer-specific mortality [12]. In our analysis, which is based on health insurance claims covering a nationwide population, we specifically examined the efficacy of metformin treatment in newly diagnosed gastric cancer patients with diabetes. The findings suggest that metformin treatment potentially delivers favorable outcomes even within this subgroup, characterized by a higher risk of mortality and morbidity. As indicated in Table 2, metformin treatment was associated with a reduction in the risks of all-cause death, disease-specific death (attributed to gastric cancer), and cardiovascular death.

Conversely, concerns surrounding the potential adverse effects of metformin usage have been raised. Among these concerns, gastrointestinal symptoms such as diarrhea, abdominal pain, dyspepsia, and constipation are the most commonly reported due to metformin’s impact on reducing hepatic gluconeogenesis and glucose uptake [38,39]. A prior study indicated that the accumulation of metformin in the intestines could be a primary cause of these gastrointestinal side effects. This may be attributed to the limited absorption of metformin hydrochloride in the stomach, with stronger accumulation occurring alongside reduced gluconeogenesis [40]. Consequently, cautious control over the drug dosage is imperative to avert the risk of severe hypoglycemia. It is important to recognize that our findings should be approached as hypothesis-generating and call for further prospective confirmation concerning the safety of long-term and/or intensive metformin treatment. While our study provides valuable insights, the potential adverse effects of metformin must be carefully weighed and investigated in dedicated studies to establish a comprehensive understanding of its safety profile.

High levels of heterogeneity are likely to emerge in pharmacological observational studies that explore the relationship between metformin usage and the risk of adverse outcomes. One factor contributing to this heterogeneity is the presence of immortal time bias, which has been a longstanding concern in observational studies [41,42]. Immortal time bias can lead to an overestimation of the benefits of a drug, potentially creating a false perception that metformin exerts a highly protective effect on morbidity and mortality risk. To mitigate the impact of immortal time bias, the present study employed a time-dependent Cox regression analysis. This approach accounted for variations in prescription patterns after the initial treatment assignment, helping to minimize bias in the estimates. Additionally, the analysis focused exclusively on individuals who had survived up to the landmark time (2~5 years) following the initial treatment assignment. This strategy was adopted to control against the potential overestimation of the drug’s effect. These rigorous methodological choices represent some of the key strengths that set this study apart from previous pharmacological observational studies. By addressing and minimizing immortal time bias, the study enhances the reliability of its findings and contributes to a more accurate understanding of the relationship between metformin and outcomes in this context.

A recent publication from the National Cancer Center highlighted temporal trends in 5-year survival rates among gastric cancer patients, revealing rates of 68.4% in 2006~2010 and 78.0% in 2016~2020 [43]. In comparison, the 5-year survival rate estimated for the overall population in our study was 69.7% (with the metformin group at 72.5% and the no-metformin group at 67.2%). Our study encompassed patients diagnosed between 2003 and 2012, with 5-year survival being assessed individually across different time periods due to variations in the date of diagnosis. Consequently, our observed rate falls within the range of the national statistics for gastric cancer survival spanning 2006 to 2020. It is worth noting that the claims data utilized in our study originated from the Korea National Health Insurance System (NHIS), a comprehensive single-payer system. This characteristic lends a high degree of external validity to our findings, bolstering the credibility and generalizability of the results.

The current study is subject to several limitations. First, the absence of detailed information regarding gastric cancer stages (e.g., stages I to IV) represents a limitation, as these data were not available. Instead, the prognosis of gastric cancer was inferred from procedural information during the initial year following diagnosis. Patients’ procedural data were categorized according to the National Comprehensive Cancer Network (NCCN) Guidelines for gastric cancer, factoring in the TNM stage (detailed in Table S4) [44]. Consequently, caution should be exercised in interpreting the findings of this study. Second, due to the nature of the National Health Insurance System (NHIS) data, only claims data were available, precluding access to medical records and limited access to health examination results. Third, it is important to acknowledge that this analysis was conducted using retrospective observational data, which inherently carries limitations such as the potential for unmeasured confounding, including drug interactions and inappropriate medication usage. Fourth, the study was unable to explore dose–response relationships. Future investigations that explore various treatment intensities could provide valuable insights for guiding safer treatment strategies for patients. Finally, the retrospective cohort design based on claims data imposes inherent limitations. Consequently, the results presented in this study cannot establish causal relationships, and residual confounding factors may persist even after employing stabilized IPTW to minimize bias. It is crucial to interpret the study’s conclusions within the context of these limitations.

## 5. Conclusions

Indeed, the study findings suggest that prolonged metformin treatment is linked to a decreased risk of all-cause death, disease-specific death (related to gastric cancer), and cardiovascular death among patients who have both diabetes and gastric cancer. This observation underscores the potential benefits of metformin therapy in improving outcomes for individuals facing this dual medical challenge.

## Figures and Tables

**Table 1:** Baseline characteristics of the study population.

Characteristics	Before Stabilized IPTW (n = 63,664)							After Stabilized IPTW (n = 63,902)						
	Metformin			No Metformin			SMD	Metformin			No Metformin			SMD
Total	29,548		(100.0)	34,116		(100.0)		29,335		(100.0)	34,567		(100.0)	
Age, years	65.4	±	9.7	64.9	±	11.1	0.051	65.2	±	9.7	65.2	±	11.0	0.001
Sex, male	20,626		(69.8)	22,992		(67.4)	0.052	20,247		(69.0)	23,931		(69.2)	0.005
Current smoker	4261		(14.4)	5752		(16.9)	0.067	4711		(16.1)	5488		(15.9)	0.005
Current drinker	2147		(7.3)	2983		(8.7)	0.054	2394		(8.2)	2823		(8.2)	0.001
Comorbidity														
Hypertension	20,326		(68.8)	18,628		(54.6)	0.295	18,020		(61.4)	21,284		(61.6)	0.003
Dyslipidemia	7908		(26.8)	13,894		(40.7)	0.299	10,019		(34.2)	11,717		(33.9)	0.005
Chronic kidney disease w/severe renal impairment ^a^	566		(1.9)	532		(1.6)	0.027	514		(1.8)	691		(2.0)	0.018
CCI	4.3	±	2.5	3.1	±	2.3	0.475	3.7	±	2.3	3.8	±	2.8	0.029
Concurrent medication														
Insulin	1209		(4.1)	139		(0.4)	0.250	627		(2.1)	874		(2.5)	0.026
OHAs	6184		(20.9)	8341		(24.5)	0.084	6996		(23.9)	8217		(23.8)	0.002
Acetylsalicylic acid	10,556		(35.7)	8945		(26.2)	0.207	9049		(30.8)	10,685		(30.9)	0.001
Statin	6861		(23.2)	5445		(16.0)	0.184	5669		(19.3)	6773		(19.6)	0.007
Procedural information ^b^														
Surgery only	16,082		(54.4)	18,805		(55.1)	0.045	15,964		(54.4)	18,810		(54.4)	0.007
Surgery/chemoradiation	4082		(13.8)	4910		(14.4)		4180		(14.2)	4971		(14.4)	
Chemoradiation	2123		(7.2)	2629		(7.7)		2295		(7.8)	2648		(7.7)	
No treatment	7261		(24.6)	7772		(22.8)		6896		(23.5)	8139		(23.5)	
Year of diagnosis														
2003~2005	7640		(25.9)	7635		(22.4)	0.082	7211		(24.6)	8607		(24.9)	0.008
2006~2009	9385		(31.8)	11,216		(32.9)		9488		(32.3)	11,184		(32.4)	
2010~2012	12,523		(42.4)	15,265		(44.7)		12,636		(43.1)	14,776		(42.7)	

Values are presented as the mean ± standard deviation or numbers (%). IPTW: inverse probability of treatment weighting; SMD: standardized mean difference; CCI: charlsons comorbidity index; OHA: oral hypoglycemic agents. ^a^ Chronic kidney disease with advanced stage requiring intensive medical therapy and financial assistance from health insurance. ^b^ Procedural information categorized based on the treatment patterns outlined in the NCCN Guidelines 2022.

**Table 2:** Result of Cox proportional hazard model for deaths after stabilized inverse probability of treatment weighting.

5 Years Endpoint	Metformin		No Metformin		Hazard Ratio (95% CI)				*p*-Value
	(n = 29,335)		(n = 34,537)						
All-cause death									
1 y	3547	(12.1)	5436	(15.7)	0.75	(0.72	–	0.78)	<0.001
2 y	5294	(18.1)	7949	(23.0)	0.76	(0.73	–	0.79)	<0.001
3 y	6385	(21.8)	9412	(27.2)	0.77	(0.75	–	0.79)	<0.001
4 y	7290	(24.9)	10,473	(30.3)	0.79	(0.76	–	0.81)	<0.001
5 y	8052	(27.5)	11,325	(32.8)	0.80	(0.78	–	0.82)	<0.001
Disease-specific death									
1 y	2791	(9.5)	4279	(12.4)	0.75	(0.72	–	0.79)	<0.001
2 y	4125	(14.1)	6165	(17.8)	0.77	(0.74	–	0.80)	<0.001
3 y	4837	(16.5)	7165	(20.7)	0.77	(0.75	–	0.80)	<0.001
4 y	5377	(18.3)	7848	(22.7)	0.78	(0.76	–	0.81)	<0.001
5 y	5780	(19.7)	8306	(24.0)	0.79	(0.77	–	0.82)	<0.001
Cardiovascular death									
1 y	533	(1.8)	773	(2.2)	0.81	(0.73	–	0.91)	<0.001
2 y	792	(2.7)	1118	(3.2)	0.83	(0.76	–	0.91)	<0.001
3 y	985	(3.4)	1355	(3.9)	0.85	(0.79	–	0.93)	<0.001
4 y	1159	(4.0)	1556	(4.5)	0.88	(0.81	–	0.94)	<0.001
5 y	1312	(4.5)	1711	(5.0)	0.90	(0.84	–	0.97)	0.004

CI: confidence interval.

## Data Availability

Data used in this study are provided in the Korea National Health Insurance Service database (https://nhiss.nhis.or.kr, accessed on 10 June 2023) and can be viewed upon reasonable request.

## Supplementary Materials

The following supporting information can be downloaded at: https://www.mdpi.com/article/10.3390/cancers15164134/s1, Figure S1. Distribution of stabilized inverse probability of treatment weighting. The density plot depicted as red color and blue color indicates metformin therapy and no metformin therapy, respectively; Figure S2. Time-to-event curve for disease-specific death; Figure S3. Time-to-event curve for cardiovascular death; Table S1. Definitions and ICD-10 codes used for adverse events; Table S2. ICD-10 codes used for Charlson comorbidity index. Table S3. Result of sensitivity analysis for death. Table S4. Types of treatment patterns according to TNM stages.
