# Supplementary material for: Association of Metformin Treatment with Risk for Death in Diabetic Patients with Concomitant Gastric Cancer
Source: Cancers (Basel). 2023 Aug 16;15(16):4134. doi: 10.3390/cancers15164134 (PMC10452498; doi:10.3390/cancers15164134)
Supplement: Supplementary file 1 [file cancers-15-04134-s001.zip › cancers-2502391-supplementary.pdf]

Supplementary Materials: Association of Metformin Treatment with Risk for Death in Diabetic Patients with Concomitant Gastric Cancer

Jae Hong Joo, Hyun-Soo Zhang, Jiyeon Chun, Eun-Cheol Park and Sohee Park

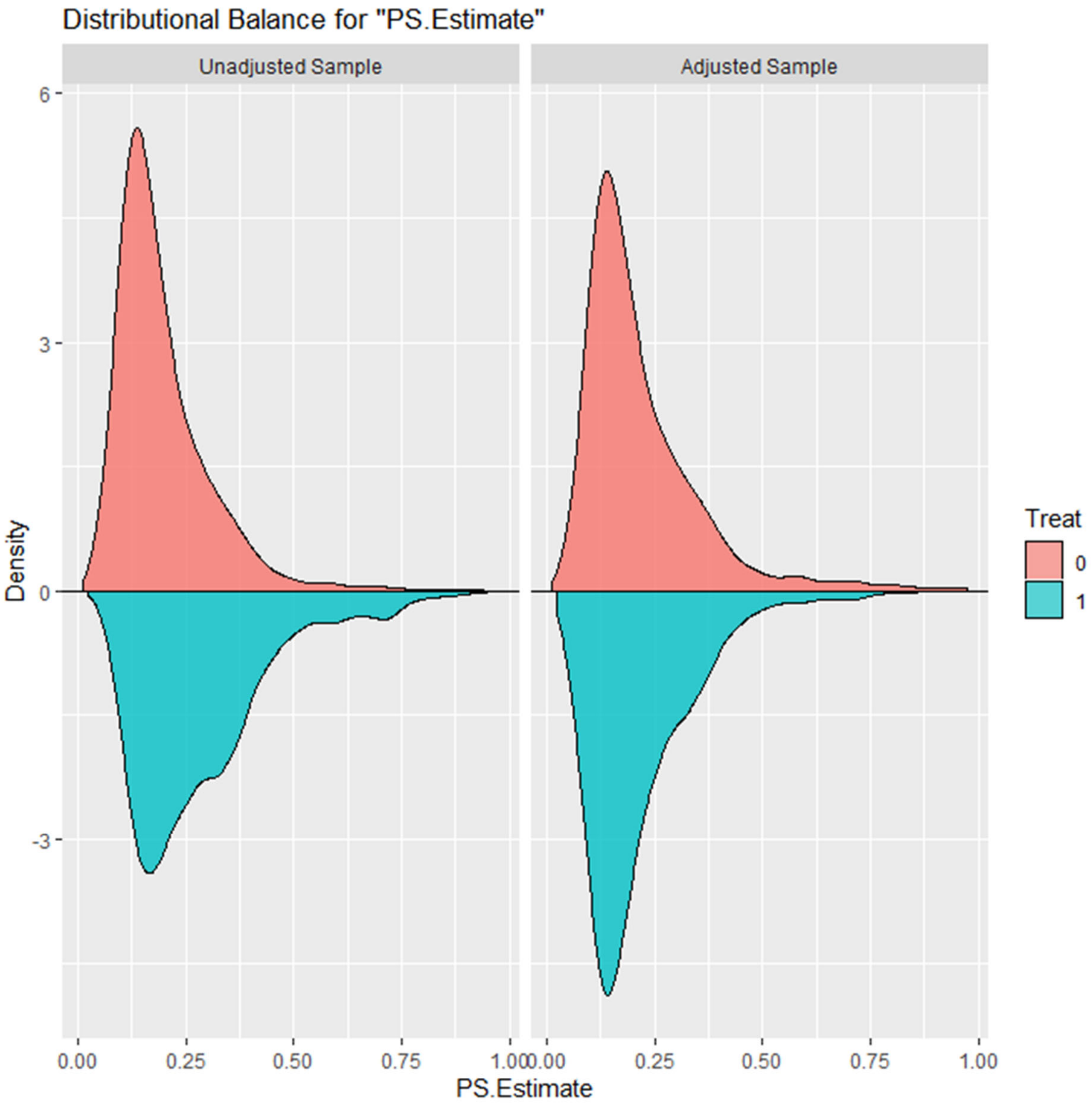

Figure S1. Distribution of stabilized inverse probability of treatment weighting. Density plot depicted as red color and blue color indicates metformin therapy and no metformin therapy, respectively.

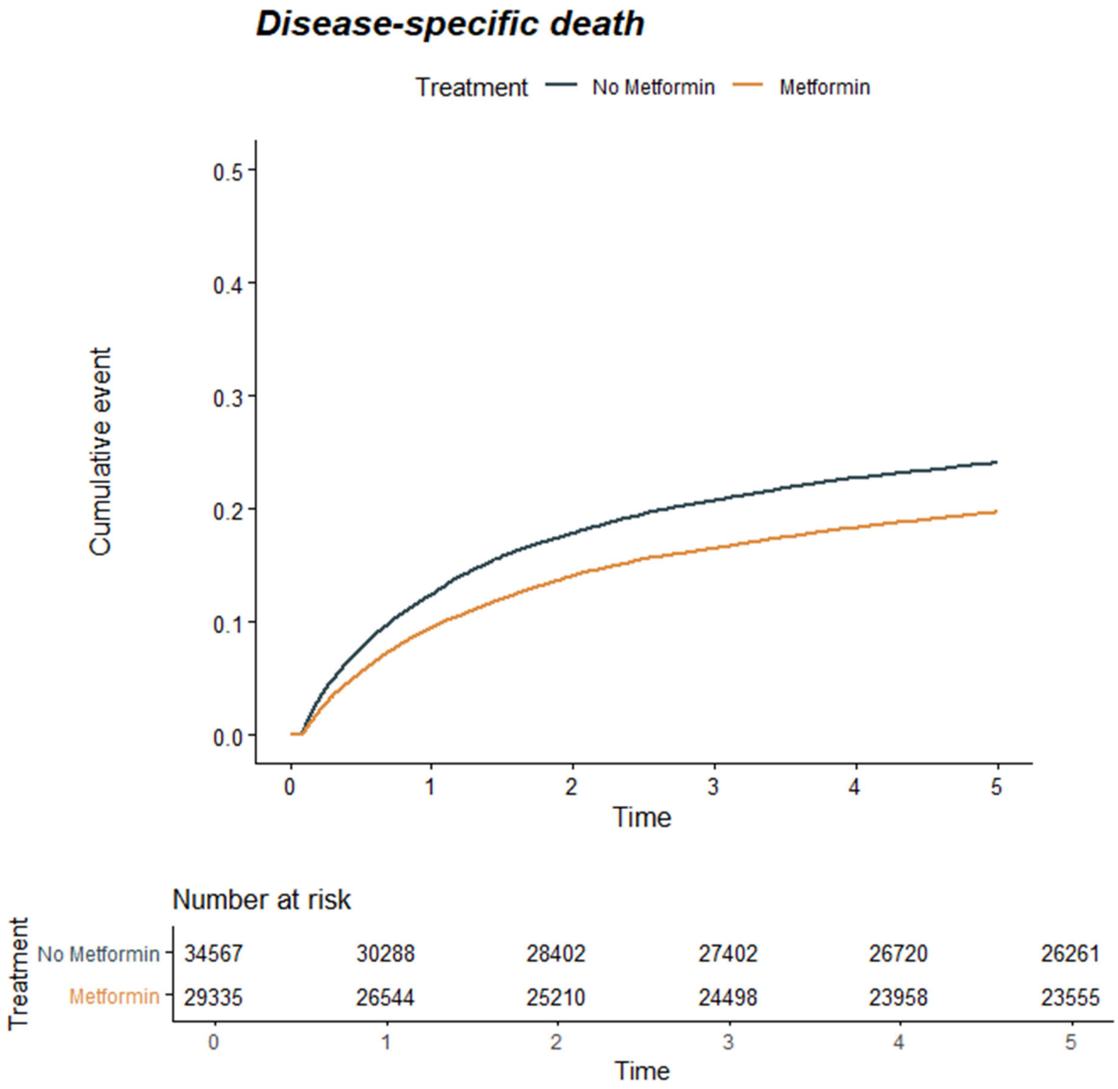

Figure S2. Time-to-event curve for disease-specific death.

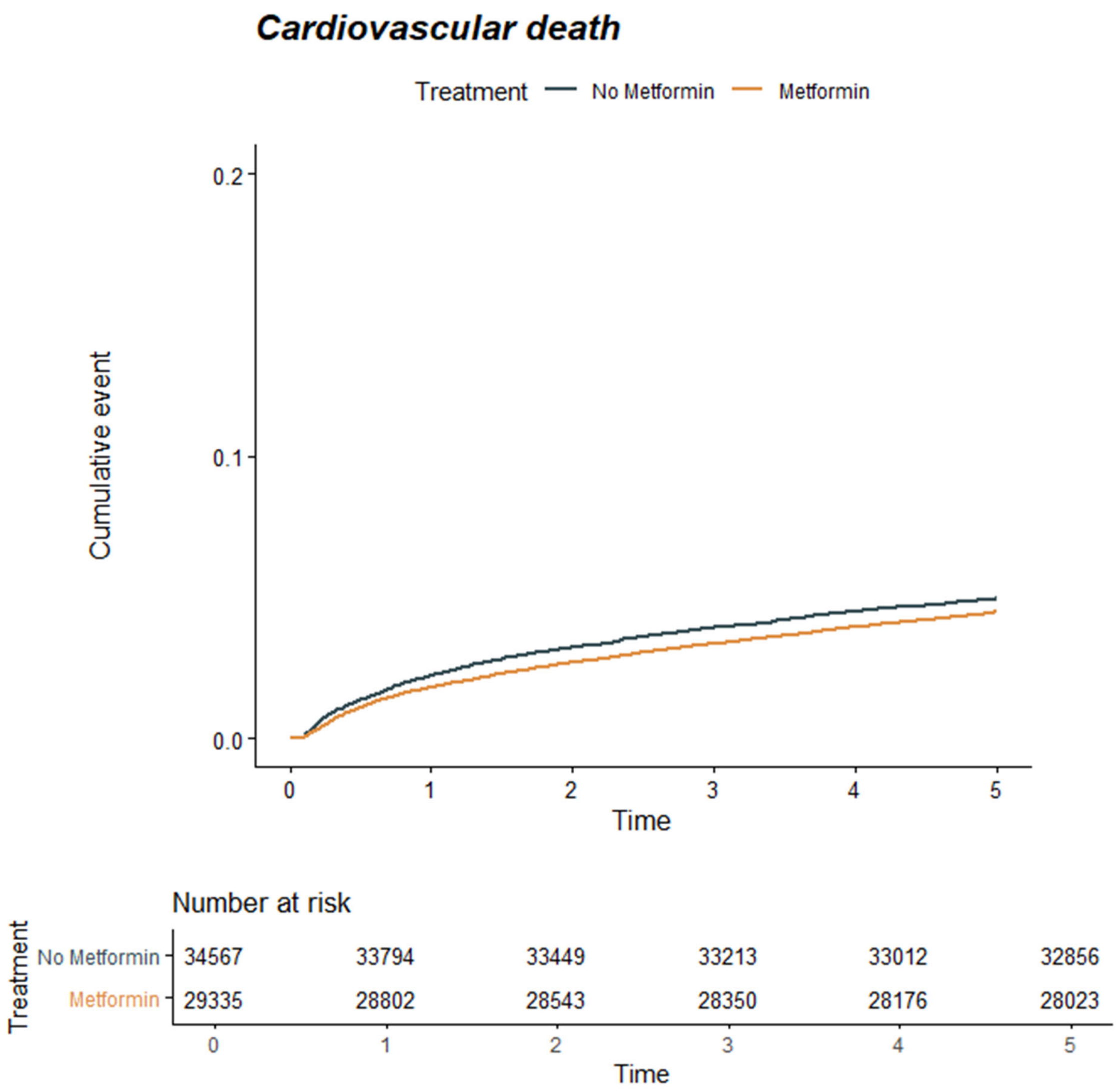

Figure S3. Time-to-event curve for cardiovascular death.

Table S1. Definitions and ICD-10 codes used for adverse events.

Clinical outcomes	Logical description and ICD-10 codes
Cardiovascular mortality	· Cardiac death confirmed by death certificate<br>· Death with ICD-10 codes corresponding to coronary artery disease, acute MI, heart failure or stroke within 1 month
Myocardial infarction (MI)	· ICD-10 codes corresponding to acute MI<br>· Performance of coronary angiography within 7 days<br>· Admission via emergency department<br>· Performance of cardiac enzyme check for more than 4 times
Hospitalization for heart failure	· Admission for more than 3 days with the following ICD-10 codes : I11.0, I50, I97.1
Non-fatal stroke	· Admission for more than 3 days with the following ICD-10 codes : I63, I64
Cancer	· ICD-10 codes corresponding to malignant neoplasms : C00-C97

ICD-10: International Classification of Diseases, Tenth Revision codes

Table S2. ICD-10 codes used for charlson comorbidity index

Comorbidities	International Classification of Diseases, 10th edition codes
Myocardial infraction	I21.x, I22.x, I25.2
Congestive heart failure	I09.9, I11.0, I13.0, I13.2, I25.5, I42.0, I42.5- I42.9, I43.x, I50.x, P29.0
Peripheral vascular disease	I70.x, I71.x, I73.1, I73.8, I73.9, I77.1, I79.0, I79.2, K55.1, K55.8, K55.9, Z95.8, Z95.9
Cerebrovascular disease	G45.x, G46.x, H34.0, I60.x-I69.x
Dementia	F00.x-F03.x, F05.1, G30.x, G31.1
Chronic pulmonary disease	I27.8, I27.9, J40.x-J47.x, J60.x-J67.x, J68.4, J70.1, J70.3
Rheumatologic disease	M05.x, M06.x, M31.5, M32.x-M34.x, M35.1, M35.3, M36.0
Peptic ulcer disease	K25.x-K28.x
Mild liver disease	B18.x, K70.0-K70.3, K70.9, K71.3-K71.5, K71.7, K73.x, K74.x, K76.0, K76.2-K76.4, K76.8, K76.9, Z94.4
Hemiplegia or paraplegia	G04.1, G11.4, G80.1, G80.2, G81.x, G82.x, G83.0-G83.4, G83.9
Moderate or severe liver disease	I85.0, I85.9, I86.4, I98.2, K70.4, K71.1, K72.1, K72.9, K76.5, K76.6, K76.7
Acquired immune deficiency syndrome/ human immunodeficiency virus	B20.x-B22.x, B24..x
Hypertension [a]	I11.X-I15.X
Renal disease [a]	I12.0, I13.1, N03.2-N03.7, N05.2-N05.7, N18.x, N19.x, N25.0, Z49.0-Z49.2, Z94.0, Z99.2

[a] Excluded in CCI calculation.

Table S3. Result of sensitivity analysis for death.

Time-dependent Cox proportional hazard model	HR	(95% CI)			
All-cause death	0.78	(0.77	-	0.79)	*
Disease-specific death	0.76	(0.75	-	0.78)	*
Cardiovascular death	0.92	(0.88	-	0.97)	
**Per-protocol population model**	**HR**	**(95% CI)**			
All-cause death	0.63	(0.60	-	0.64)	*
Disease-specific death	0.62	(0.61	-	0.64)	*
Cardiovascular death	0.73	(0.71	-	0.75)	*

*P-value < 0.001.

Table S4. Types of treatment pattern according to TNM stages

Abbrev.	Treatment name	TNM stages
s	Surgery alone	Tis, T1a, or T1b with any N
s-d±r	-	T1 with N1 or T2 with N0
d-s-d	Perioperative chemotherapy	T2 with any N
d±r-s	Preoperative chemoradiation	T2 with any N
s-d	Postoperative chemotherapy	T3-T4 with any N, or any T with N+
s-d-d±r-d	Postoperative chemoradiation	T3-T4 with any N, or any T with N+, all with residual dis. after surgery
d±r	(Definitive) chemoradiation	Unresectable or metastatic dis.(M+)
d	Systemic therapy	Unresectable or metastatic dis.(M+)
none	Best supportive care	Unresectable or metastatic dis.(M+)

s: surgery, d: drug(chemotherapy), r: radiation therapy, d±r: chemoradiation

(Source: NCCN. Guidelines for Gastric Cancer. 2022.)
